# Metabolic reprogramming of the urea cycle pathway in experimental pulmonary arterial hypertension rats induced by monocrotaline

**DOI:** 10.1186/s12931-018-0800-5

**Published:** 2018-05-11

**Authors:** Hai-Kuo Zheng, Jun-Han Zhao, Yi Yan, Tian-Yu Lian, Jue Ye, Xiao-Jian Wang, Zhe Wang, Zhi-Cheng Jing, Yang-Yang He, Ping Yang

**Affiliations:** 1 Department of Cardiology, China–Japan Union Hospital of Jilin University, Changchun, China; 2 State Key Laboratory of Cardiovascular Disease, FuWai Hospital, and Key Laboratory of Pulmonary Vascular Medicine, Peking Union Medical College and Chinese Academy of Medical Sciences, Beijing, China; 3 State Key Laboratory of Bioactive Substance and Function of Natural Medicines, Institute of Materia Medica, Peking Union Medical College and Chinese Academy of Medical Sciences, Beijing, China

**Keywords:** Pulmonary arterial hypertension, Metabolomics, Urea cycle, Pathway, Biomarker

## Abstract

**Background:**

Pulmonary arterial hypertension (PAH) is a rare systemic disorder associated with considerable metabolic dysfunction. Although enormous metabolomic studies on PAH have been emerging, research remains lacking on metabolic reprogramming in experimental PAH models. We aim to evaluate the metabolic changes in PAH and provide new insight into endogenous metabolic disorders of PAH.

**Method:**

A single subcutaneous injection of monocrotaline (MCT) (60 mg kg^− 1^) was used for rats to establish PAH model. Hemodynamics and right ventricular hypertrophy were adopted to evaluate the successful establishment of PAH model. Plasma samples were assessed through targeted metabolomic profiling platform to quantify 126 endogenous metabolites. Orthogonal partial least squares discriminant analysis (OPLS-DA) was used to discriminate between MCT-treated model and control groups. Metabolite Set Enrichment Analysis was adapted to exploit the most disturbed metabolic pathways.

**Results:**

Endogenous metabolites of MCT treated PAH model and control group were well profiled using this platform. A total of 13 plasma metabolites were significantly altered between the two groups. Metabolite Set Enrichment Analysis highlighted that a disruption in the urea cycle pathway may contribute to PAH onset. Moreover, five novel potential biomarkers in the urea cycle, adenosine monophosphate, urea, 4-hydroxy-proline, ornithine, N-acetylornithine, and two candidate biomarkers, namely, O-acetylcarnitine and betaine, were found to be highly correlated with PAH.

**Conclusion:**

The present study suggests a new role of urea cycle disruption in the pathogenesis of PAH. We also found five urea cycle related biomarkers and another two candidate biomarkers to facilitate early diagnosis of PAH in metabolomic profile.

## Background

Pulmonary arterial hypertension (PAH) is a rare and devastating disease characterized by progressive pulmonary vascular remolding, which ultimately leads to right ventricle (RV) failure and death [1, 2]. Major advances have been achieved in the understanding of pathobiology and treatment of PAH; however, the disease remains to be an incurable condition associated with substantial morbidity and mortality. The 5- and 7-year survival rates for patients with PAH are 57 and 49%, respectively [3, 4].

PAH is increasingly being recognized as a systemic disorder associated with substantial metabolic dysfunction [5, 6]. Recent studies have demonstrated the relationship of the metabolic syndrome with PAH and highlighted the features of insulin resistance [7], adiponectin deficiency [8], dyslipidemia [9], fatty acid oxidation, and the tricarboxylic acid cycle [10] in the development of pulmonary vascular disease. The complex pathobiology of PAH involves various metabolic pathways related to inflammation, oxidative stress, plaque composition, and lipid metabolism, ultimately leads to endothelial damage, increased pulmonary vascular resistance, and right heart failure [10]. Improved understanding of the specific metabolic pathobiology of PAH is critical in exploring the pathogenesis of PAH and uncovering the novel therapeutic targets for this devastating disease.

Metabolomics targets the extensive characterization and quantitation of small molecular metabolites from exogenous and endogenous sources and has emerged as a novel avenue for advancing precision medicine [11]. Recent evidence has shown the abnormalities of small molecular metabolites in patients with PAH [12] and has led to the emergence of numerous metabolomic studies on PAH. Yidan et al. reported disrupted glycolysis, upregulated tricarboxylic acid cycle, and increased fatty acid metabolite production with altered oxidation pathways in patients with severe PAH [13]. Lewis et al. also reported the plasma metabolite biomarkers of PAH, indoleamine 2,3-dioxygenase, and the association with RV–pulmonary vasculature dysfunction [14]. These studies suggested that metabolomics is a powerful tool for the examining the pathology, prevention, diagnosis, and therapy of PAH.

In the present work, we used integrated targeted metabolomics to detect lipids and polar metabolites from only 100 μl of a biosample. A monocrotaline (MCT)-induced rat model was used to identify the metabolic profiles of PAH with the integrated targeted metabolomic strategy. The potential biomarkers found in PAH rat plasma may facilitate earlier PAH detection and a thorough understanding of the PAH mechanism.

## Methods

### Animal experiment

MCT-induced animal model was used to assess PAH development in rats. All experiments were conducted in accordance with the Guideline for Care and Use of Laboratory Animals published by the US National Institutes of Health (NIH publication 85–23, revised 1996) and approved by the Institutional Committee for Use and Care of Laboratory Animals of FuWai Hospital (Beijing, China).

Sprague–Dawley rats (180–220 g, 6 weeks old) were provided by Vital River Laboratories Co., Ltd. (Beijing, China). A total of 15 male rats were housed under specific pathogen-free conditions (12 h light/12 h dark photoperiod, 25 ± 2 °C, 50% ± 5% relative humidity) and were allowed to acclimate for 2 weeks before experiments. Rats were divided randomly into two groups: the PAH model group received a single subcutaneous injection of MCT (60 mg/kg; Sigma, St. Louis, MO, USA, *n* = 7), whereas the control group (*n* = 8) was treated with saline. After 3 weeks, all the rats were weighed and anesthetized (chloral hydrate, 60 ml/kg, *n* = 15).

### Hemodynamic analysis and right ventricle hypertrophy (RVH) assessment

To examine the development of PAH, we measured the mean pulmonary artery pressure (mPAP), right ventricular systolic pressure (RVSP), and RVH. For right heart catheterization, a polyethylene catheter was inserted into the right external jugular vein and threaded into the RV and pulmonary artery to measure the mPAP and RVSP. All data were analyzed using the PowerLab data acquisition system (Power Lab 8/30; AD Instruments, Sydney, Australia). The RV free wall was removed from the left ventricle (LV) and septum. RVH was accessed by the weight ratio of the RV to the LV plus septum weight (RV/(LV + S)).

### Immunofluorescence staining and histological analyses

The rats were euthanized and dissected after catheterization. Following PBS perfusion, lung tissues were embedded in 4% formaldehyde for immunofluorescence staining or in 10% formalin for histological analyses. The tissues were cut into 5 μm-thick slices. Anti-α-smooth muscle actin (α-SMA, 1:300, Abcam) was incubated at 4 °C overnight and then with Alexa 488 conjugated anti-rat IgG at room temperature for 1 h. Slides were viewed with a fluorescence microscope (LSM 780, Carl Zeiss, Oberkochen, Germany). Double-blind quantitative analysis was adopted to evaluate both vascular thickness and muscularization level. To analyze the degree of pulmonary vascular remodeling, ten random visual fields of wall area/total vessel area and relative fluorescence intensity were analyzed per lung section at a magnification of 200 using ImageJ software (http://rsbweb.nih.gov/ij).

### Sample collection and preparation

The blood samples were collected from the euthanized rats by using EDTA as anticoagulant to obtain plasma by centrifugation (3000 rpm, 15 min, 4 °C) and then maintained at − 80 °C. The plasma was thawed at 4 °C and re-homogenized through brief vortex mixing. Then, 100 μl of plasma was transferred into a 1.5 ml Eppendorf tube and combined with 20 μl of sphingolipid internal standards and 20 μl of polar metabolite internal standards. After the mixture was vortexed for 10 s, 400 μl of acetonitrile was added to the tube. The sample was vortexed for 5 min, allowed to stand for another 15 min, and then centrifuged at 13000 rpm for 10 min (4 °C). Protein precipitation was removed, and the supernatant was transferred into another glass tube and evaporated under a nitrogen stream (room temperature). The organic residue was then redissolved with 100 μl of acetonitrile/methanol (75:25, *v*/v) for polar metabolite analysis followed by ultrasonication. The aliquots were consequently vortexed for 10 min and transferred into a 1.5 ml Eppendorf tube. After centrifugation for 10 min (13,000 rpm, 4 °C), the supernatant was transferred to a UPLC–MS/MS auto sampler vial.

Rigorous method validation of polar metabolites was established before metabolomics analysis to ensure the accurate and reliable of the analytical method, such as linearity and lower limit of quantification, precision and accuracy, stability, replaceable matrix and carryover (published in our previous work) [15]. To ensure the accuracy of the analysis, pool sample and pool standard solution were used as quality control in the whole analytical batches. The metabolites with compound relative standard deviation less than 30% between pool sample and pool standard sample were further analysis.

### Instrument conditions

Experiments were performed using an Agilent 6490 Triple Quadrupole LC–MS apparatus. A Waters XBridge Amide column (2.1 mm × 100 mm, 3.5 μm particle size; Waters, Milford, MA, USA) was used for chromatographic separation. The column temperature was 35 °C. Mobile phase A comprised acetonitrile/water (50:50, *v*/v) containing 15 mM ammonium acetate in water containing 0.2% ammonium hydroxide. Mobile phase B comprised acetonitrile/water (95:5, v/v) containing 15 mM ammonium acetate in water containing 0.2% ammonium hydroxide. The gradient was programmed as follows: 0–10 min, 100% B; 10–23 min, 100–0% B; 23–24 min, 0–100 %B; and 24–30 min, 100% B. The flow rate was 0.3 ml/min, and the injection volume was 5 μl.

The parameters for AJS electrospray ionization MS/MS in positive/negative ion mode were as follows: dry gas: nitrogen; dry gas temperature, 200 °C; dry gas flow rate, 14 l/min; nebulizer, 20 psi; sheath gas: nitrogen; sheath gas temperature, 250 °C; sheath gas rate, 11 l/min; capillary voltage, ± 3000 V and nozzle voltage, ± 1.5 kV. Multiple reaction monitoring was performed using the characteristic precursor-to-product ion transitions, fragmentor voltage (380 V), and collision energies. The polar metabolites were identified based on retention time by using authentic standards and quantified through standard curve samples.

### Statistical analysis

A t-test was used to compare between two groups for normal distribution data or Mann–Whitney test for non-normal distribution data by using SPSS 18.0 software (SPSS Inc., Chicago, IL, USA). A *p* value of less than 0.05 was considered significant. To identify the most significant metabolites involved in the pathophysiology of PAH, we used MetaboAnalyst 3.0, a useful online website, to explore the potential metabolite and the involved pathway [16]. For further data analysis, partial least squares discriminant analysis (PLS-DA) was used to visually discriminate between groups by using the SIMCA-P 14.1 software (Umetrics, Umeå, Sweden). To reduce the noises and artifacts of the metabolomic data, all measured concentrations were mean-centered and auto-scaled. The quality and predictability of the PLS-DA model was then evaluated by R2Y (cum) and Q2 (cum) values, respectively. Metabolite Set Enrichment Analysis was conducted to identify biologically meaningful patterns significantly enriched in the quantitative metabolomic data.

## Results

### Establishment of PAH model

**Fig. 1:**
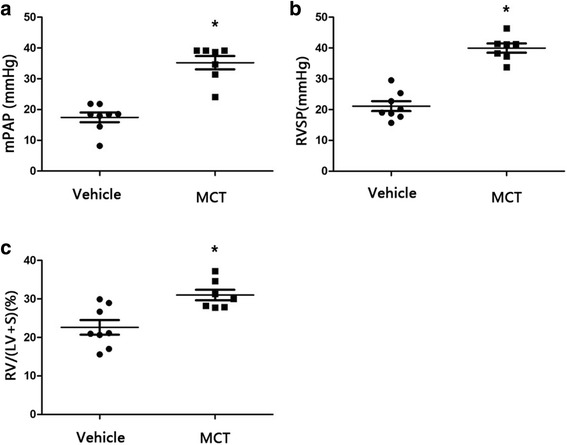
Successful establishment of PAH model in MCT-treated group. The mPAP (**a**), RVSP (**b**), RV/(LV + S) (**c**) were significantly higher in the MCT group than that in vehicles (MCT, *n* = 7; Vehicle, *n* = 8). MCT = monocrotaline; mPAP = mean pulmonary artery pressure; RVSP = right ventricular systematic presure; RV/(LV + S) = right ventricular/(left ventricular + septum) (*P < 0.05)

**Fig. 2:**
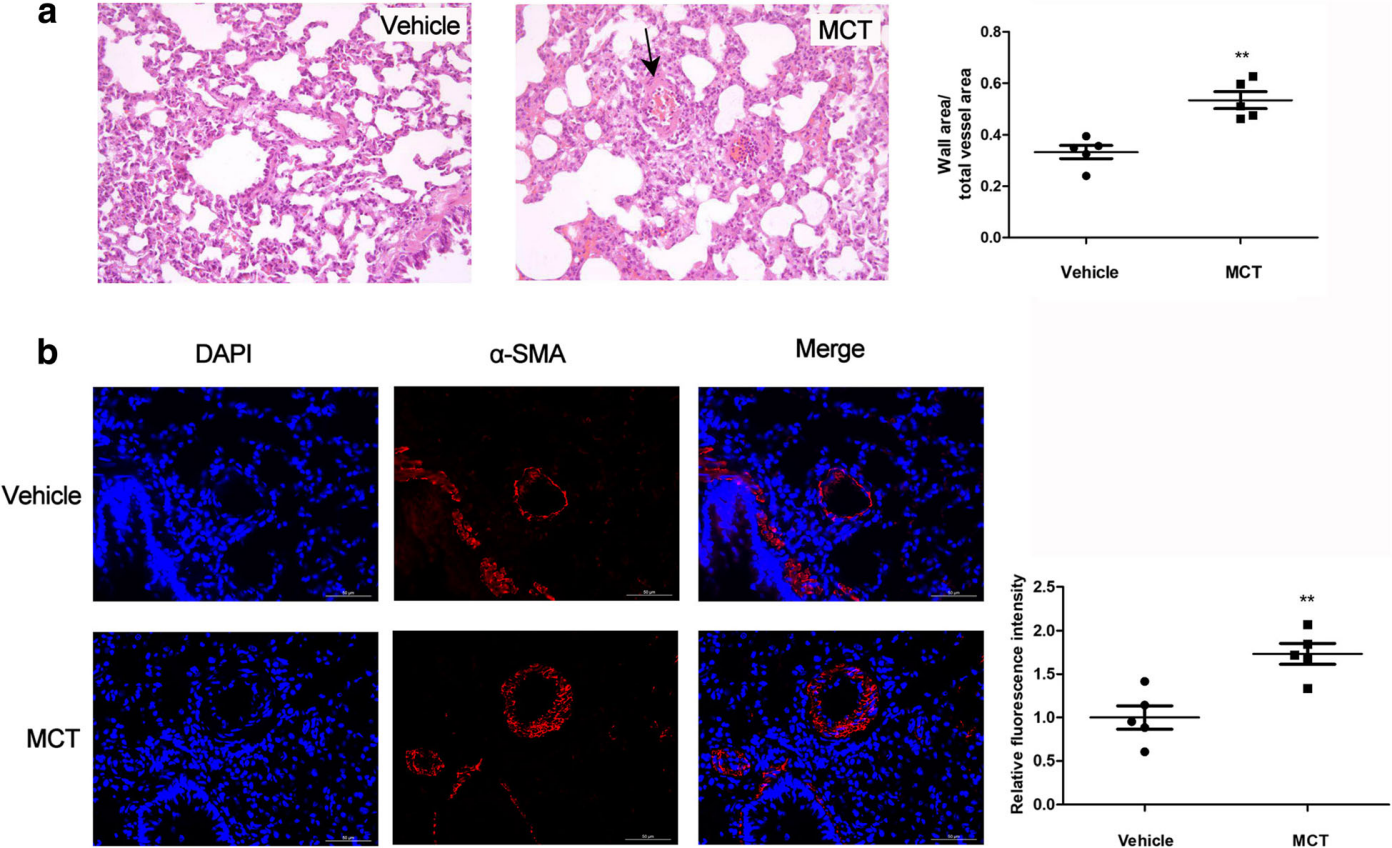
Increased pulmonary vascular remodeling in MCT induced rats. (**a**) Representative micrographs of histological assessment demonstrating thickening of the pulmonary vascular (black arrow) in the PAH model group; (**b**) Representative micrographs of Immunostaining of lung-tissue-treated rats revealing increasing α-SMA expression in the distal pulmonary arteries (MCT, *n* = 5; Vehicle, *n* = 5). α-SMA = α-smooth muscle actin; MCT = monocrotaline (**P < 0.01)

PAH is characterized by a sustained increase in pulmonary artery pressure and vascular remolding associated with pulmonary arteriole obliteration [17]. In the present study, the MCT-treated rats (*n* = 7) exhibited dramatically elevated mPAP (35.22 ± 5.75 vs. 17.45 ± 4.41, *p* < 0.001) and RVSP (39.97 ± 3.96 vs. 21.11 ± 4.53, *p* < 0.001) than those of the control group (*n* = 8) (Fig. 1a, b). MCT-treated rats also developed pronounced RVH evident by the drastic increase in RV/LV + S (31.01% ± 3.65% vs. 22.61% ± 5.34%, *p* < 0.05) (Fig. 1c). In addition, histological assessment demonstrated increased proliferation of the pulmonary vascular and the immunostaining of MCT-treated lung tissue showed increased α-SMA expression in the distal pulmonary arteries in the PAH model group relative to that in the control rats (Fig. 2a, b). These results indicated the successful establishment of the PAH model in our analysis.

### Metabolomics study

Plasma samples (100 μl) were analyzed using the targeted metabolomic profiling platform. In total, 126 polar metabolites were quantified from the MCT-treated and control rat plasma. Unpaired t test and Mann–Whitney test were performed to determine the metabolite variations between the two groups. Thirteen plasma metabolites related to PAH were tentatively identified through the targeted metabolomic pattern analysis to be significantly altered between the MCT-treated and control groups (*p* < 0.05). The detailed information of the distinguished metabolites was summarized in Table 1. The metabolites were ranked by significance on the basis of the *p* values. Our results demonstrated that many metabolites involved in different metabolic pathways were altered in rat plasma after MCT treatment.

**Table 1:** Differential metabolites between PAH model and controls

Name of metabolites	Category	Fold change	P value	VIP
AMP	Nucleotides	0.033	1.76E-05	2.127
urea	Organic acids	1.407	0.007	2.108
O-acetylcarnitine	lipid	1.321	0.007	1.859
cytosine	Organic compounds	1.643	0.007	2.004
2′-dexycytidine	Nucleosides	1.606	0.007	1.880
indole	Organic carbonic acids	1.438	0.013	1.658
betaine	Organic acids	1.408	0.014	1.625
p-hydroxybenzoate	others	1.417	0.019	1.830
N-acetylornithine	Organic acids	1.401	0.019	1.816
ornithine	Organic acids	1.417	0.036	1.790
4-hydroxy-L-proline	Organic acids	1.374	0.038	1.357
dc-SMA	others	1.734	0.051	1.492
phenylacetylglycine	Organic acids	3.2318	0.053	1.777

*VIP* variable importance in projection, *AMP* adenosine monophosphate

Thirteen differential metabolites were divided into five categories: organic acids (*n* = 7), nucleotides (*n* = 2), lipid (*n* = 1), organic compounds (*n* = 1) and “others” (*n* = 2), which comprised the materials that cannot clearly be classified into any of the other four categories. The organic acids accounted for the largest proportion of the metabolites. Among the 13 differential metabolites, only adenosine monophosphate (AMP) was significantly decreased in the PAH group than in the control group. The AMP concentration in the PAH group was only 0.03-fold of the control group. The rest of the differential metabolites (92.3%) in the PAH group were all elevated relative to those in the control group. In particular, phenylacetylglycine increased by 3.23 folds that in the control group (Table 1).

### Targeted metabolomic pattern analysis

**Fig. 3:**
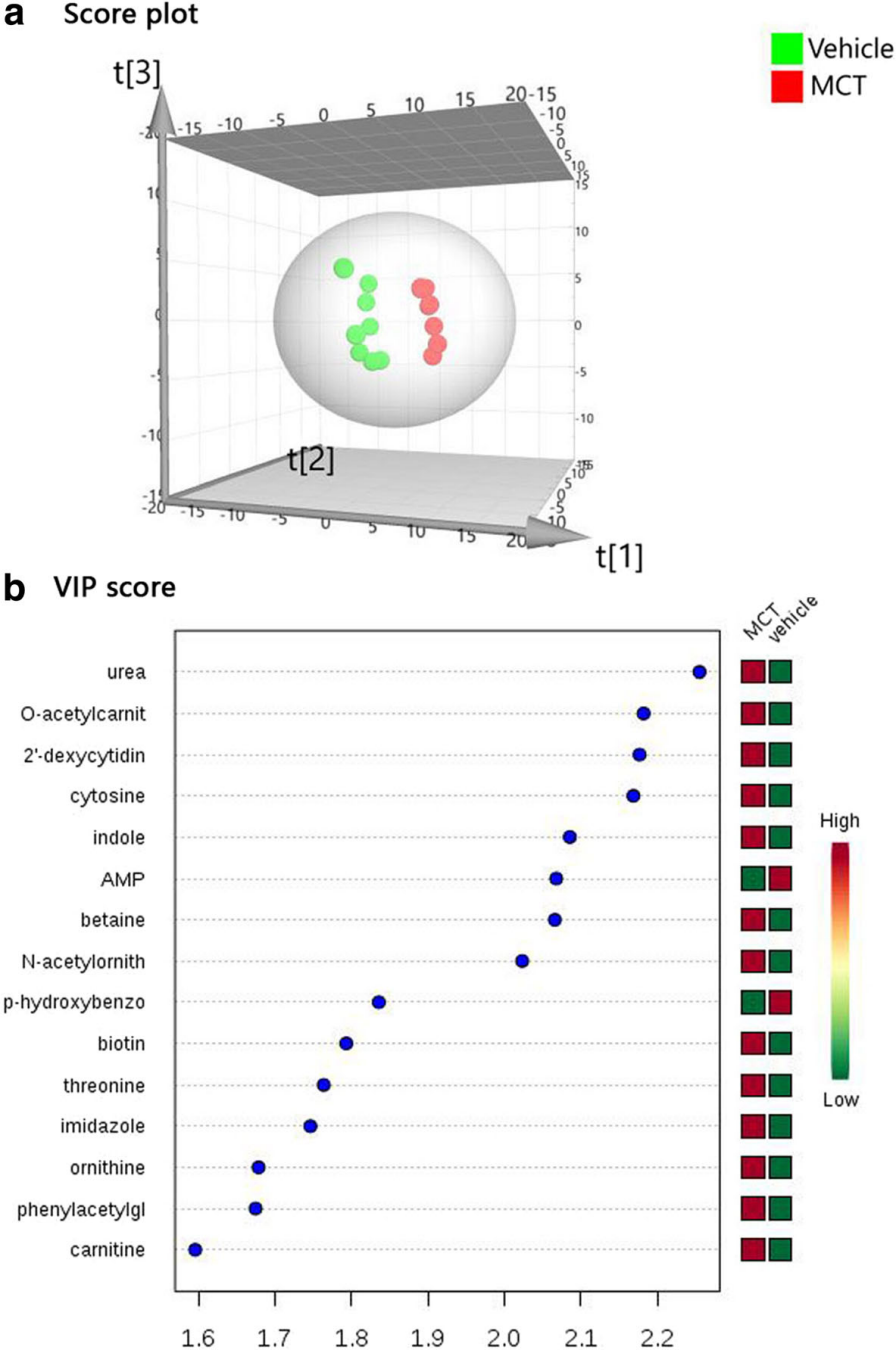
Distinctive Metabolomic profiling of pulmonary hypertension. **a** Score plot obtained from principal component analysis exhibited a distinct metabolic signature between MCT-treated group and Vehicle. **b** VIP score and related concentration of the differential metabolites (VIP score > 1.5). AMP = adenosine monophosphate; MCT = monocrotaline; VIP = variable importance in projection

PLS-DA, a supervised method based on a partial least squares algorithm, shows a high sensitivity for biomarker detection [18]. In this study, PLS-DA was conducted to investigate the metabolite patterns of PAH model and control group. The score plot obtained though PLS-DA revealed that the PAH model aggregated to the right side, whereas the control group clustered to the left (Fig. 3a). There was a distinguished classification between the clustering of the PAH model and control groups in the plasma with R2Y and Q2 greater than 0.5, which suggested that the PLS-DA models showed good stability and predictability. Those results indicated that the differentially expressed metabolites can be used to separate the plasma samples into two distinct groups.

**Fig. 4:**
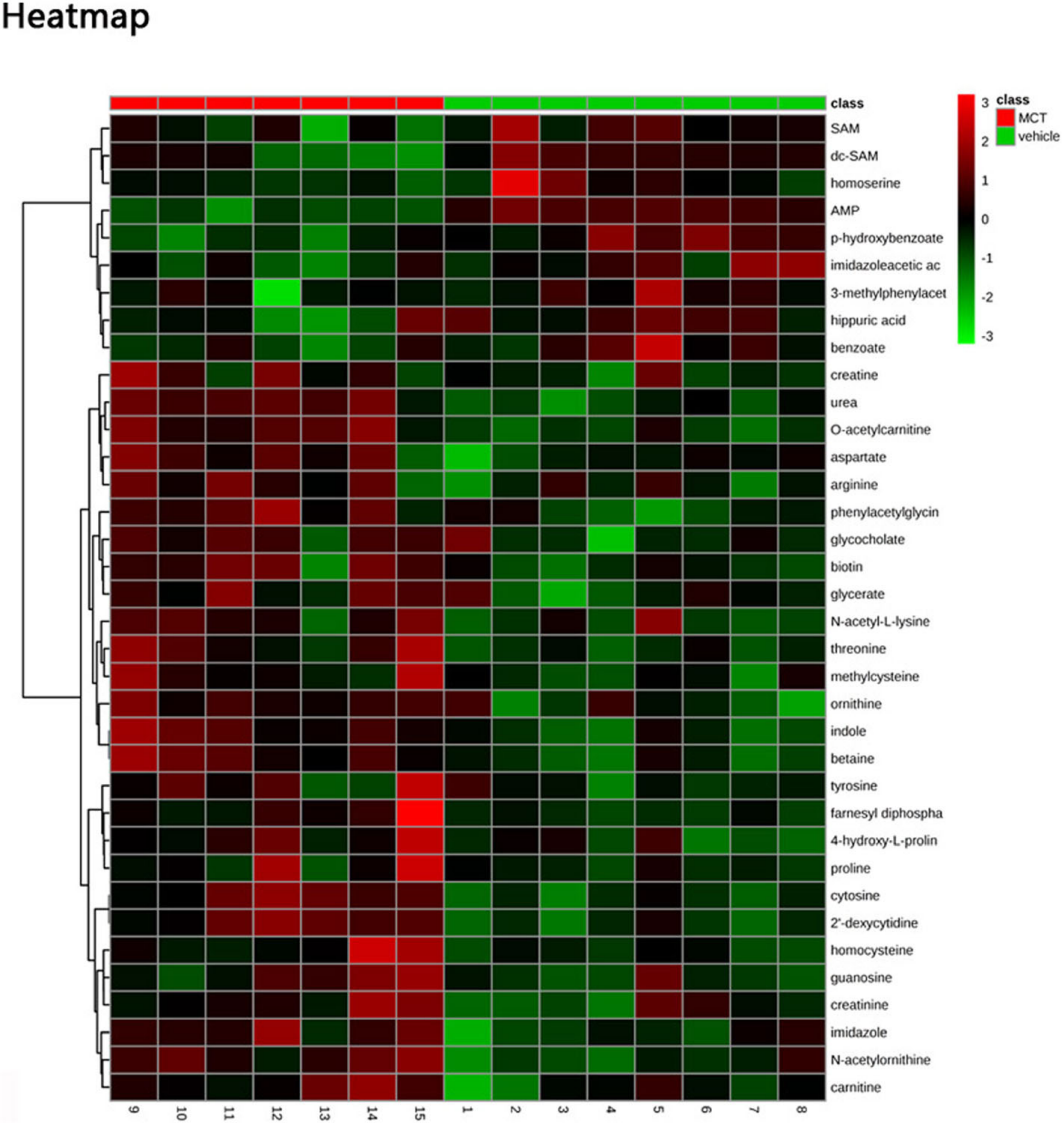
Heat map of the differential metabolites. Representative the non-supervised hierarchical clustering of VIP score top 36 differential metabolites in Principal component analysis relative to vehicle sample data (MCT, *n* = 7; Vehicle, *n* = 8). Shades of light (red/green) represent the increase and decrease of a metabolite, respectively, relative to the median metabolite levels. MCT = monocrotaline

We then identified differential metabolites for class discrimination between the groups based on the variable importance in projection (VIP) score obtained from PLS-DA. A total of 15 differential metabolites features identified by PLS-DA were presented in the Fig. 3b (VIP score > 1.5). The VIP score and relative concentrations of the corresponding metabolite in each group were also presented. The distinguished metabolic features were ranked by significance on the basis of their specific VIP values. Most of the (84.6%, 11/13) metabolites obtained from unpaired t test were included in the 15 differential metabolites. These multiple metabolic changes reflected an important metabolic distinction of PAH in the heat map based on non-supervised hierarchical clustering (VIP score top 36, Fig. 4). Overall, the PAH plasma exhibited a distinct metabolic signature relative to that in the control group.

### Metabolite set enrichment analysis

**Fig. 5:**
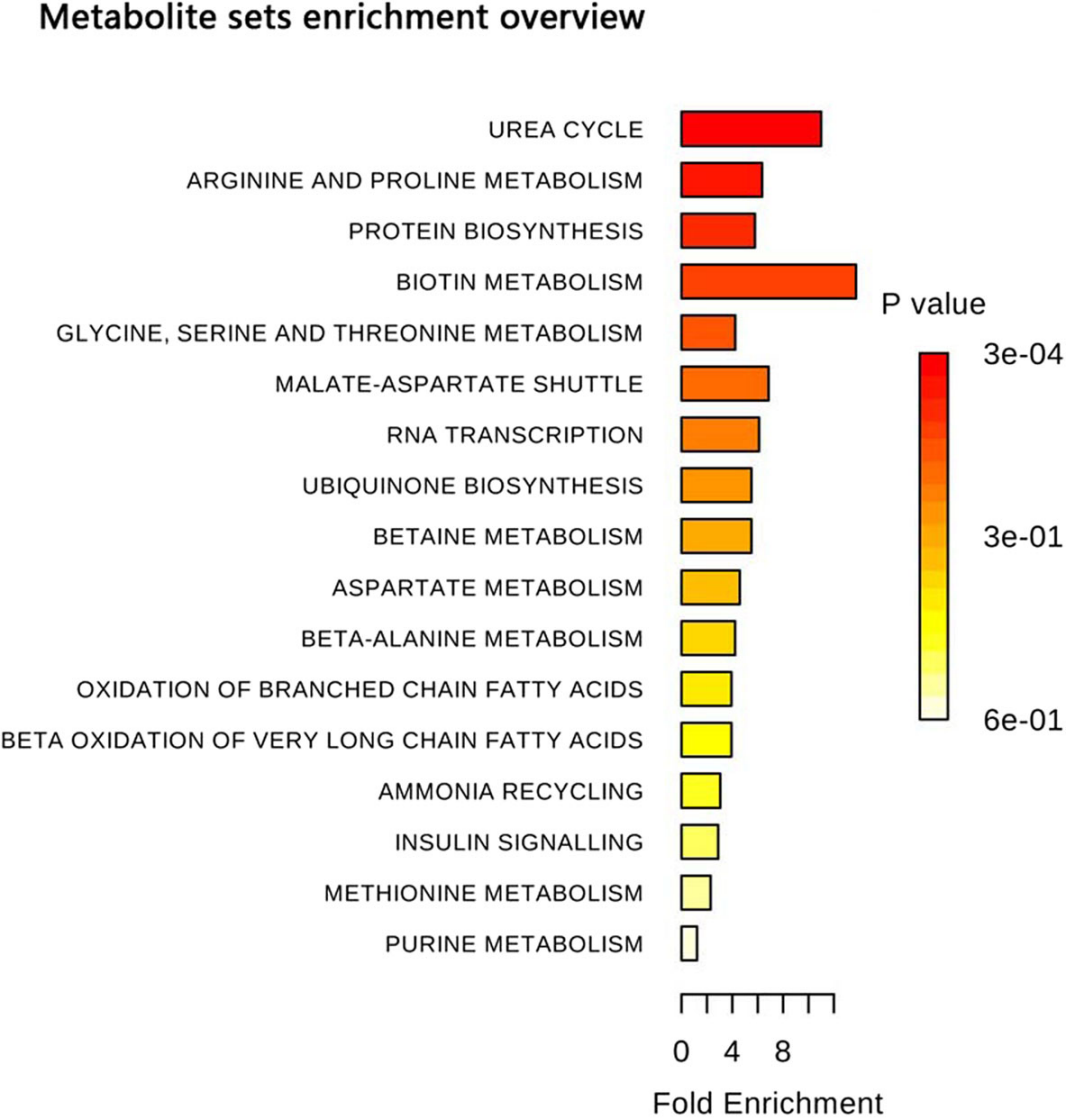
The results of Metabolite Set Enrichment Analysis

Over representation analysis is a method that uses a hypergeometric test to evaluate whether a particular metabolite set is represented more than expected by chance within a given compound list. Differential metabolites and their concentrations were imported to MetaboAnalyst 3.0 to exploit the most disturbed metabolic pathways via over representation analysis. The metabolites that discriminate PAH were involved in 17 pathways (Fig. 5). After the results were adjusted for multiple testing by using one-paired *p* value, only the urea cycle pathways were enriched with the metabolites of interest (*p* = 0.02).

**Fig. 6:**
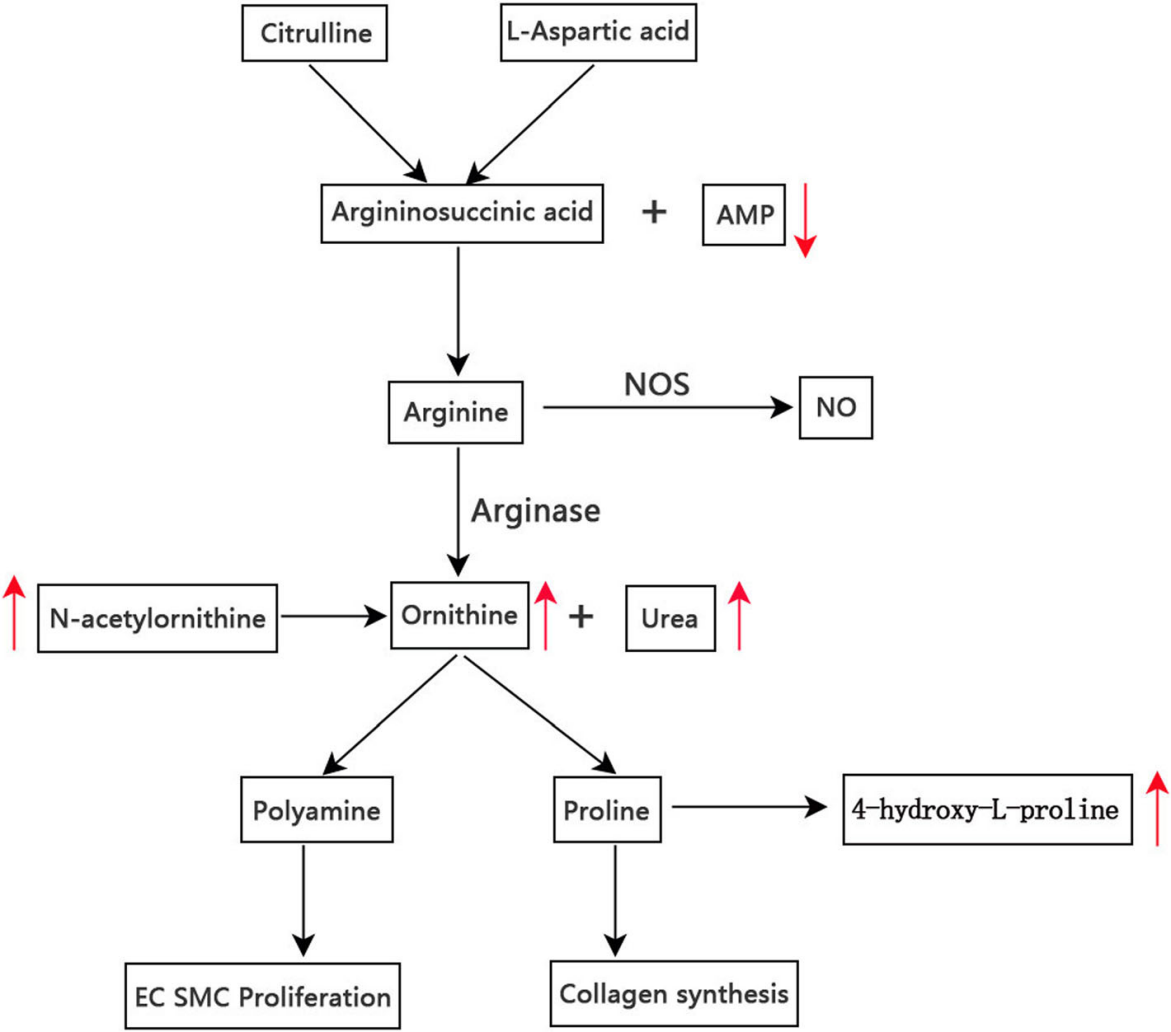
Pathways of urea cycle disturbance in PAH. PAH = pulmonary artery hypertension; AMP = adenosine monophosphate; EC = endothelial cell; NO = nitric oxide; NOS = nitric oxide synthase; SMC = smooth muscle cell

**Fig. 7:**
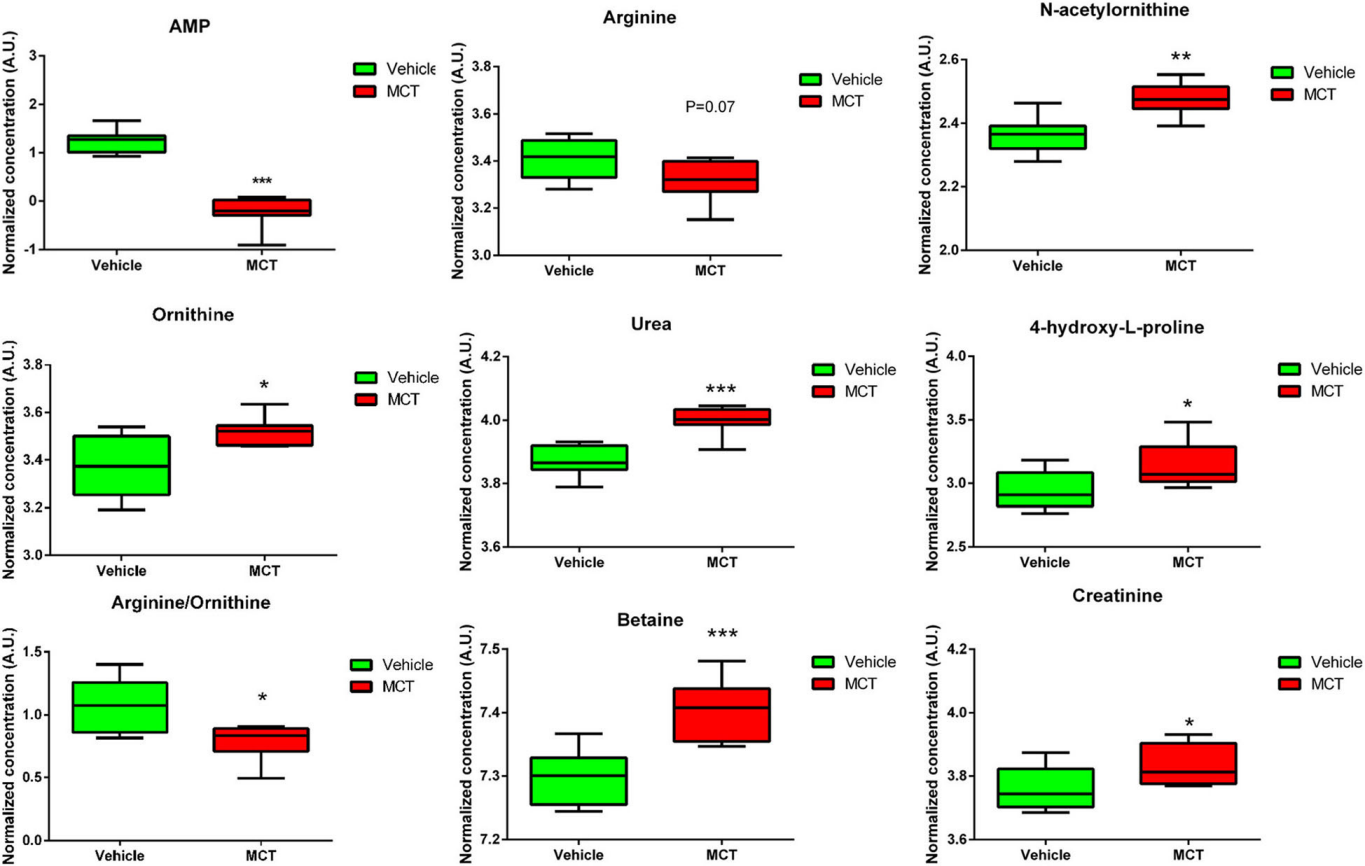
Metabolite profiles of main differential metabolites obtained from the quantitative analysis of the subjects

Figure 6 shows the related urea cycle pathway from the KEGG and SMPDB. Urea cycle pathway, playing a major role in PAH severity and treatment response [19, 20], connected five major distinguished metabolites in this study. These metabolites were AMP, 4-hydroxy-proline, ornithine, urea, and N-acetylornithine which demonstrated great potential in differentiating the PAH group from the control group (*p* < 0.05, VIP score > 1). The corresponding metabolite profiles are shown in Fig. 7. Citrulline and aspartic acid are synthesized to AMP and arginosuccinic acid, which is then converted to arginine by argininosuccinate lyase. Arginine is the precursor of nitric oxide (NO); nitric oxide synthase (NOS) converts arginine to citrulline while simultaneously producing NO and water. Arginine can also be converted to ornithine and urea by arginase. N-acetylornithine can be converted to ornithine by the aminoacylase-1. Ornithine is then converted to polyamines and proline, which are involved in the proliferation of pulmonary arterial smooth muscle cells and collagen synthesis and contribute to the pathogenesis of PAH. Proline then can be converted to 4-hydroxy-proline by Prolyl 4-hydroxylase. These compounds are considered as candidate biomarkers because of their significant ability to differentiate the PAH model from the control, as demonstrated in this study. These results suggest that the disruption of the urea cycle pathway may contribute to PAH onset.

## Discussion

In this study, we used a target metabolomic platform to analyze 126 metabolites in plasma from rats treated with either MCT or saline. A total of 13 differential metabolites involved in urea cycle, arginine and proline metabolism, protein biosynthesis, and glycine metabolism were identified. Our results demonstrated that the MCT-treated PAH model was marked by a pattern of global metabolomic heterogeneity distinct from that in the saline-treated control. Further enrichment analysis highlighted the urea cycle as the most disturbed metabolic pathway contributing to the pathogenesis of PAH. Moreover, five novel potential biomarkers in the urea cycle, namely, AMP, ornithine, 4-hydroxy-proline, urea, and N-acetylornithine, and two candidate biomarkers, namely, O-acetylcarnitine and betaine, were found as potential biomarkers highly correlated with PAH in our study. Our results open an avenue for earlier PAH detection and improve the understanding on target metabolic pathway alterations in the progression of PAH.

NO, a critical factor in cell growth and vasodilation has been well profiled in the pathogenesis of PAH [21–23]. The substrate of NO is arginine, which is mainly supplied by the urea cycle, is a linkage of the urea cycle to PAH [24]. NOS converts arginine to citrulline while simultaneously producing NO and water [25]. The utilization of arginine by other enzymes, particularly arginase, decreases the availability of arginine for reaction with NOS. Arginase, the enzyme that converts arginine to ornithine and urea, can compete with NOS for arginine leading to a decreased NO expression [26] (Fig. 6).

The mechanism underlying the reduced NO bioavailability in PAH involves the factors regulating NOS activity, i.e., substrate arginine and arginase expression and activity [27]. The increased arginase activity or expression competes with NOS and hence induces a decreased arginine and NO production in PAH patients [28, 29]. A lower arginine-to-ornithine ratio, which indicates a higher arginase activity, was found to be associated with greater severity and mortality in PAH (risk ratio: 2.5; 95% confidence interval: 1.2, 5.2, *p* = 0.006) [27, 30, 31]. Additional, previous studies revealed that the substrate arginine levels were decreased and inversely related to pulmonary artery pressure [26, 27, 32]. Our analysis also shown a decreased trend of arginine in the PAH model than control group (*p =* 0.07). Furthermore, we found an increased urea expression (1.4-fold change than control) and a decreased arginine-to-ornithine ratio (*p* < 0.05) (Fig. 7) in MCT-treated group, which further confirmed the results of previous works [29–31, 33].

N-acetylornithine is another circulating metabolite involved in the urea cycle. It is a minor component of deproteinized human blood plasma. N-acetylornithine is converted to ornithine by the aminoacylase-1. Meanwhile, ornithine is a precursor of polyamines and proline, which are involved in cell proliferation and collagen synthesis, respectively (Fig. 6) [26]. Proline then can be converted to 4-hydroxy-proline. In our analysis, both the expression of N-acetylornithine and 4-hydroxy-proline was significantly increased in PAH model than those found in the control group. The increased 4-hydroxy-proline indicated increased metabolic level of proline although polyamines and proline were not directly detected in our study. We hypothesize that increased N-acetylornithine leads to increased ornithine metabolism to proline, which may contribute to the proliferation of pulmonary arterial smooth muscle cells [33]. The increased N-acetylornithine induced an upregulated ornithine/proline pathway, which may contribute to a hyperproliferative phenotype in the PAH model.

AMP, an intermediary substance of the adenosine triphosphate (ATP) involved in energy metabolism, is also an important component of the urea cycle. Citrulline and aspartic acid are synthesized to arginosuccinic acid with ATP transform to AMP. Arginosuccinic acid is a precursor of arginine in the urea cycle/citrulline–NO cycle (Fig. 6). In this study, the AMP expression was only 0.3-fold that in the control group and this expression level may cause arginosuccinic acid deficiency, interrupt the citrulline–NO cycle, and further decrease NO expression.

Additional, adenosine monophosphate-activated protein kinase (AMPK) is a highly conserved serine/threonine protein kinase that plays an important role in vascular homeostasis and is involved in the pathogenesis of PAH [34]. AMPK exerts a pro-apoptotic effect in vascular smooth muscle cells [35] and an anti-apoptotic effect in endothelial cells [36]. AMP is a direct sensor activated by AMPK through binding to the gamma subunit; this occurrence triggers the phosphorylation of the catalytic alpha subunit and may hence further exacerbate the pathogenesis of PAH [37]. Teng et al. demonstrated that AMPK activity and expression were decreased in pulmonary artery endothelial cells. Metformin, an AMPK activator, increases the bioavailability of NO and restores angiogenesis in pulmonary artery endothelial cells [34]. AMPK activation also significantly reduces RVSP and RVH and inhibits the pulmonary artery remolding in the MCT-induced rat PAH model [38]. All these results imply that AMPK may play a protective role in PAH, and the decreased AMP levels in the PAH group may adversely affect the AMPK and consequently aggravate the phenotype of the disease.

Some of the other metabolic abnormalities detected in our analysis have been reported as potential biomarkers for early PAH diagnosis in previous studies. Betaine is a methyl donor in the formation of methionine, which is vital for protein synthesis in pulmonary arterial smooth muscle cell proliferation [39, 40]. In our study, the betaine level was significantly higher in the PAH group than in the control group (*p* < 0.05). Increased betaine may lead to abnormal mitochondrial structure and function and result in energy metabolism disorders [41]. Acetylcarnitine is an acetic acid ester of carnitine that facilitates the movement of acetyl CoA into the mitochondria during fatty acid oxidation. Brittan et al. found that the circulating fatty acid long-chain acylcarnitines are elevated in patients with PAH and are associated with fatty acid accumulation in the myocardium caused by reduced fatty acid oxidation [42]. High acylcarnitine levels were detected in our analysis and are consistent with previous study results [42]. In future studies, a group of biomarkers reflecting different pathways dysregulated in pulmonary vascular disease, including the NO pathway, mitochondrial bioenergetics, and fatty acid oxidation, can provide a comprehensive insight into the pathogenesis of PAH.

In the present study, we adopted a feasible, accurate, and robust targeted metabolomic profiling platform that can simultaneously extract and quantify 126 metabolites covering the core network of lipid, energy, amino acid, and nucleotide metabolism from the same microamount of biological sample. Our results simultaneously highlighted the metabolic pathways dysregulated in PAH and provided new insight into the involvement of the urea cycle in the pathogenesis of PAH. However, the sample size in this study was relatively small. Further study utilizing a larger sample size and plasma or lung tissue samples from human PAH patients are needed to validate the present findings.

## Conclusions

In summary, we used a targeted metabolomic profiling platform to show a disrupted urea cycle pathway with increased urea, N-acetylornithine, and ornithine levels, 4-hydroxy-proline and decreased AMP metabolite levels in the plasma of a MCT-induced PAH model. Our results enabled the further understanding of the role of a disrupted urea cycle in the pathogenesis of PAH and also found five urea cycle related biomarkers and other two candidate biomarkers to facilitate early diagnosis of PAH in metabolomic profile.

## Abbreviations

AMP: Adenosine monophosphate
AMPK: Adenosine monophosphate-activated protein kinase
ATP: Adenosine triphosphate
LV: Left ventricle
mPAP: mean pulmonary artery pressure
NO: Nitric oxide
NOS: Nitric oxide synthase
PAH: Pulmonary artery hypertension
PLS-DA: Partial least squares discriminant analysis
RV: Right ventricle
RVH: Right ventricle hypertrophy
RVSP: Right ventricular systolic pressure
S: Septum
VIP: Variable importance in projection
α-SMA: α-smooth muscle actin
